# Hydrodynamic characteristics of the two-phase flow field at gas-evolving electrodes: numerical and experimental studies

**DOI:** 10.1098/rsos.171255

**Published:** 2018-05-02

**Authors:** Cheng-Lin Liu, Ze Sun, Gui-Min Lu, Jian-Guo Yu

**Affiliations:** 1 National Engineering Research Center for Integrated Utilization of Salt Lake Resource, East China University of Science and Technology, Shanghai, People's Republic of China; 2 State Key Laboratory of Chemical Engineering, East China University of Science and Technology, Shanghai, People's Republic of China

**Keywords:** computational fluid dynamics, experimental measurements, gas-evolving electrodes, particle image velocimetry, volumetric three-component velocimetry

## Abstract

Gas-evolving vertical electrode system is a typical electrochemical industrial reactor. Gas bubbles are released from the surfaces of the anode and affect the electrolyte flow pattern and even the cell performance. In the current work, the hydrodynamics induced by the air bubbles in a cold model was experimentally and numerically investigated. Particle image velocimetry and volumetric three-component velocimetry techniques were applied to experimentally visualize the hydrodynamics characteristics and flow fields in a two-dimensional (2D) plane and a three-dimensional (3D) space, respectively. Measurements were performed at different gas rates. Furthermore, the corresponding mathematical model was developed under identical conditions for the qualitative and quantitative analyses. The experimental measurements were compared with the numerical results based on the mathematical model. The study of the time-averaged flow field, three velocity components, instantaneous velocity and turbulent intensity indicate that the numerical model qualitatively reproduces liquid motion. The 3D model predictions capture the flow behaviour more accurately than the 2D model in this study.

## 1. Introduction

Gas-evolving vertical electrode system is a typical electrochemical industrial reactor [1]. In these gas–liquid flow systems, gas bubbles drag the surrounding electrolyte with them, which induces a circulation of the bulk liquid between the electrodes. The flow field in these systems is inherently unsteady because of the strong coupling between the gas and liquid phases. The bubbles strongly affect the liquid flow field pattern and local electrolyte mixing near the electrodes [2,3]. To improve the efficiency of gas–liquid electrochemical reactors, the hydrodynamics in the two-phase system must be thoroughly understood. A general description of the gas-evolving vertical electrodes and the modelling state of these systems was given by Vogt [4]. Many experiments [5–10] have been performed to collect experimental data about the hydrodynamics in the gas-evolving vertical electrodes. Boissonneau & Byrne [11] investigated the effects of the bubble size, gas rate and channel width on the liquid velocity in a small rectangular cell with vertical electrodes using microscope-enhanced visualization, laser Doppler velocimetry (LDV) and particle image velocimetry (PIV). The results showed that the flow field in the system with bubble evolution could be transformed from laminar to turbulent. Ali & Pushpavanam [12] used PIV to investigate liquid circulation in an alkaline electrolysis cell with various electrode designs and under various voltage and concentration operating conditions.

The number of available commercial computational fluid dynamics software programs has increased tremendously in recent years, and the calculation ability of workstations or even desktop PCs is sufficient to overcome the multi-field coupling problems. In the field of gas-evolving vertical electrodes, massive studies of fluid dynamics in systems have been carried out because a reduction in physical testing and analysis by mathematical simulations results in substantial time and cost savings [13]. Mat & Aldas [14,15] used PHOENICS to create a two-fluid model for the gas–liquid flow in different gas-evolving electrochemical reactors and found that the current density and bubble size can affect the gas release and the liquid-phase flow. Wedin & Dahlkild [16] developed a numerical model to investigate the effects of bubble size and channel width on the electrolyte flow in natural convection. Dahlkild [17] also simulated the effect of the gas distribution along a single vertical gas-evolving electrode by solving the boundary-layer equations using a finite-difference method. Caspersen & Kirkegaard [18] developed an analytical model to calculate the gas-phase distribution and cell conductivity in an electrolysis cell under natural convection. Philippe *et al*. [19] investigated the hydrodynamics of the vertical electrode in a laboratory-scale electrolysis cell using a numerical simulation. Sun *et al*. [20] developed a three-dimensional (3D) mathematical model to describe the flow field in an electrolysis cell and used PIV data collected for an electrolysis cell of zinc sulfate to validate the model. Tsuge *et al*. [21] investigated the effects of the electrolyte velocity and the average current density on the current density distribution in a horizontal electrolysis cell. Alexiadis and co-workers [22–24] investigated the liquid–gas flow patterns in single and multiple narrow gas-evolving channels using a Euler–Euler two-phase model with OpenFOAM. They noted that the flow pattern could transfer into ‘pseudo-turbulent’ if the current density was high and a sufficient amount of small bubbles was present.

The instantaneous measurement of 3D velocity fields is essential for hydrodynamics analysis. Conventional experimental observation techniques such as Pitot tubes, hot-wire anemometers and LDV [25,26] have been widely used by previous researchers. However, all of these techniques share a common limitation of only one-point measurement per time unit. PIV [27–29] typically uses a single camera to simultaneously obtain two components of the fluid velocity on a plane. The accuracy of the experimental measurements is affected by the out-of-plane velocity component because the information describing the out-of-plane motions is embedded in the in-plane motion [30]. Therefore, the desired measurement technique should be capable of capturing 3D structures to obtain a comprehensive description of the 3D flow. Stereoscopic PIV [31,32], holographic PIV [33], tomographic PIV [34,35], defocusing digital PIV [36], 3D particle tracking velocimetry [37,38] and volumetric three-component velocimetry (V3V) [39,40] can be used to measure the 3D flow. V3V is a new non-intrusive visualization experimental technique that enables the instantaneous measurement of the 3D flow in a volume with a maximal depth of 100 mm and cross-sectional size of 120 mm × 120 mm.

In the present work, we used the PIV and V3V techniques to quantify the two-dimensional (2D) and 3D flow structures in a cold model of a gas-evolving vertical-electrode system. Furthermore, the corresponding 2D and 3D mathematical models were studied using an Eulerian–Eulerian approach of COMSOL. The objective of this work was comparing the experimental measurements obtained using PIV and V3V with the results of an Eulerian–Eulerian model, and to investigate the hydrodynamic characteristics of the two-phase flow field at gas-evolving electrodes, including the three orthogonal velocity components and the turbulent intensity distribution on the basis of the instantaneous and time-averaged velocity fields.

## 2. Experimental and mathematical methods

### 2.1. Experimental apparatus and methods

**Figure 1.**
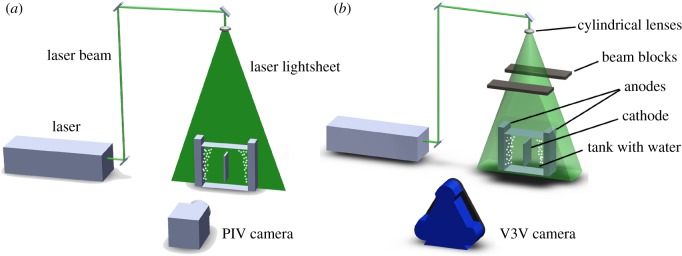
Schematic of the experimental set-up: (*a*) PIV and (*b*) V3V.

All experiments were conducted in a rectangular glass cell (200 × 185 × 100 mm^3^) filled with water to a depth of 140 mm. A schematic of the experimental system is shown in figure 1. Two organic polymethylmethacrylate plates were placed vertically on two sides of the cell as the anodes, and a plastic plate was placed in the centre to represent the cathode. Two vertical working areas, which were made of 110 × 100 mm^2^ porous glass plates with a pore size of approximately 50 µm were embedded in the vertical working area of the two anodes and submerged in liquid. Bubbles with sizes ranging from 50 µm to 2 mm were generated on the porous glass plate surfaces by compressed air forced through the porous glass plates [41–43].

The flow field was first measured using a 2D PIV system (PowerView Plus, TSI Inc., USA). The beam of an Nd:YAG laser (Continuum Inc., USA) passed through an optical arrangement of lenses, which generated an approximately 1 mm-thick laser sheet, and illuminated the measurement region from the top. The PIV system was assembled with only one frame-straddling digital CCD camera with a resolution of 4 million pixels, which was mounted perpendicular to the illumination light sheet. A schematic of the PIV system is shown in figure 1*a*. The interrogation areas for the cross correlation were 64 × 64 pixels with 50% overlap. In the image-capture component of the V3V apparatus, the camera probe (TSI Inc., USA) uses three digital CCD cameras to view the flow field from three perspectives, and each camera has a high resolution of 4 million pixels. As shown in figure 1, cameras were also positioned in front of the cell, and their distance was approximately 700 mm. The measurement was conducted using the same Nd:YAD laser with an energy of 200 mJ pulse^−1^. The laser beam was spread in the *x*- and *z*-directions after passing through an optical arrangement lens. Two beam blocks were placed at the edge of the cell to limit the beam thickness (100 mm) to illuminate only the measurement volume.

In the experimental cell, water was seeded with polycrystalline tracer particles with a mean diameter of approximately 50 µm. Laser sequencing and the CCD camera frames were triggered twice in a time interval of 2000 µs using a laser pulse synchronizer. The displacement of particles in the time interval was recorded at a rate of 7 Hz for almost 70 s.

**Figure 2.**
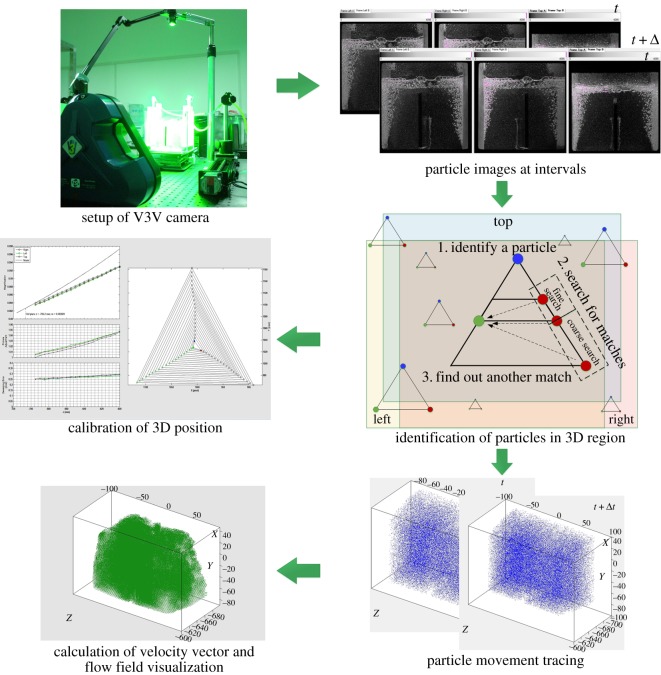
Experimental procedure of the V3V measurement.

A single V3V capture includes three image pairs, whereas a PIV capture includes only one image pair. The image processing of V3V, which includes particle detection, particle tracking and velocity field interpolation, was performed using the Insight V3V 4G software. Instantaneous velocity fields were determined in four steps: identification of 2D particle locations from each of three apertures, determination of 3D particle locations in space, tracking individual particles in the volume and interpolating the resulting randomly spaced vectors onto Cartesian coordinates. The entire experimental procedure of the V3V measurement is shown in figure 2. The velocity vector field may contain spurious vectors because of noise that arises during the acquisition of bubbles substantially larger and brighter than the tracer particle. The rising velocity of 1 mm bubbles was approximately 0.3 m s^−1^, which is significantly larger than the liquid velocity in the experiment [44]. Therefore, a filter with a range of ±0.2 m s^−1^ for the three-component velocities was applied to the raw data to eliminate the bubble vectors and obvious spurious vectors.

### 2.2. Mathematical models and simulations

The present problem can be considered a bubbly flow, which is typically encountered in bubble column reactors, nuclear industry or other industrial applications [45–47]. Two-phase flow models can be classified into two main types: Euler–Lagrangian and Euler–Euler models [48]. In this work, an Euler–Euler model was selected because high-volume fractions must be treated, which is trickier with Euler–Lagrange models. To ensure a manageable problem, the model was subject to the following assumptions:

(1) The fluids in both phases are viscous, incompressible and Newtonian.
(2) The two phases share the same pressure field.
(3) The physical properties of the cell material are constant.
(4) The flow is assumed to be isothermal, so the energy equation is not required.
(5) The bubble–bubble interaction is incorporated in the drag force.
(6) No bubble coalescence or break-up occurs, and the bubble size can be considered approximately constant.

#### 2.2.1. Governing equations

With these assumptions, the Euler–Euler approach considers both liquid and gaseous phases with certain volume fractions [49]. The following equations are used:

2.1 $$\frac{\partial \alpha_{i}\rho_{i}}{\partial t}+\nabla \cdot (\alpha_{i}\rho_{i}u_{i})=0$$

and

2.2 $$\frac{\partial \alpha_{i}\rho_{i}u_{i}}{\partial t}+\nabla \cdot (\alpha_{i}\rho_{i}u_{i}u_{i})=-\nabla (\alpha_{i}p)-\nabla (\alpha_{i}\tau_{i})+\alpha_{i}\rho_{i}g+F_{i},$$

where *α_i_* is the void fraction of phase *i*. The sum of the void fractions of all phases is equal to 1. *ρ* is the density; *p* is the pressure; *F_i_* is the interphase forces between the phases per unit volume, which couples the two phases of the equations; *τ_i_* is the stress tensor and is described as follows:

2.3 $$\tau_{i}=-\mu_{\mathrm{eff},i}\left(\nabla u_{i}+{\left(\nabla u_{i}\right)}^{T}-\frac{2}{3}I{\left(\nabla u_{i}\right)}^{T}\right),$$

where $\mu_{\mathrm{eff},i}$ is the effective viscosity, and *I* denotes the identity tensor. For the liquid phase, the effective viscosity is the sum of the dynamic viscosity $\mu_{L,i}$ and turbulent viscosity $\mu_{T,i}$:

2.4 $$\mu_{\mathrm{eff},i}=\mu_{L,i}+\mu_{T,i}.$$

The turbulence properties, which include the turbulent kinetic energy *k* and the dissipation rate of turbulent kinetic energy *ε*, were solved using the standard *k-ε* model.

2.5 $$F_{i}=F_{i}^{\mathrm{drag}}+F_{i}^{\mathrm{td}}.$$

$F_{i}^{\mathrm{drag}}$ is the most important force discussed here and is defined by

2.6 $$F_{i}^{\mathrm{drag}}=-\frac{3}{4}\rho_{l}\frac{\pi d_{b}^{2}}{4}C_{D}|u_{l}-u_{g}|(u_{l}-u_{g}),$$

where *d*_b_ is the bubble diameter and *C*_D_ is the drag coefficient, which can be mathematically defined by semi-empirical relations. In the current computation, the drag coefficient depends on the bubble Reynolds number *Re*_b_ as follows [50]:

2.7 $$C_{D}=\left\{ \begin{array}{ll} \frac{24}{Re_{b}}(1+0.15Re^{0.687}) & Re_{b}\leq 1000\\ 0.44 & Re_{b}\geq 1000. \end{array}\right.$$

The turbulent dispersion force $F_{q}^{\mathrm{td}}$ is based on the analogy with molecular movement, as derived by Lopez de Bertodano [51]. Some authors have claimed that this effect is important for industrial flows [52,53]:

2.8 $$F_{q}^{\mathrm{td}}=-C_{\mathrm{td}}\rho_{q}k_{q}\nabla \alpha_{c},$$

where the value of coefficient $C_{\mathrm{td}}$ is suggested to be 0.09–0.1.

#### 2.2.2. Boundary conditions

No-slip boundary conditions are applied for all phases at the solid walls:

2.9 $$u_{l}=u_{g}=v_{l}=v_{g}=0.$$

At the anode active area, a gas inlet is defined, and a no-slip condition is used for the liquid phase. The gas flux at the anode is

2.10 $${\text{v}}_{g}=\frac{Q}{A},$$

where *Q* is the mass flow rate of the gas evolution, and *A* is the anode active area.

The interface between the electrolyte and air is modelled with zero shear for the liquid phase, and the gas phase is assumed to flow outward from the free surface at the rate at which it reaches the surface.

2.11 $$u_{g}=u_{g}$$

and

2.12 $$u_{l}\cdot n=0.$$

For the initial condition, a velocity of 0 m s^−1^ and a pressure of 1 bar was applied. Recent computations to handle the gas-evolution process were performed using a constant bubble size [15,24,54]. A bubble diameter of 1.0 mm was used in the mathematical model; the bubble diameters in the experiments were measured. Other physical properties of the fluid are given in table 1.

**Table 1.** Physical properties of the fluid phase.

*ρ*_l_ (kg m^−3^)	*ρ*_g_ (kg m^−3^)	*μ*_l_ (kg m^−1^ s^−1^)	*μ*_g_ (kg m^−1^ s^−1^)
998	1.22	1.00 × 10^−3^	1.75 × 10^−5^

#### 2.2.3. Solution method

These governing equations with the boundary conditions were solved with the standard solver of COMSOL Multiphysics. A time step size of 5 × 10^−3^ s was used for all simulations. For each time step, the absolute tolerance was set as 10^−5^. These simulation parameters and procedures were used in both 2D and 3D models. The velocity profile was extracted and compared with the experimental results.

## 3. Results and discussion

### 3.1. Reliability validation

**Figure 3.**
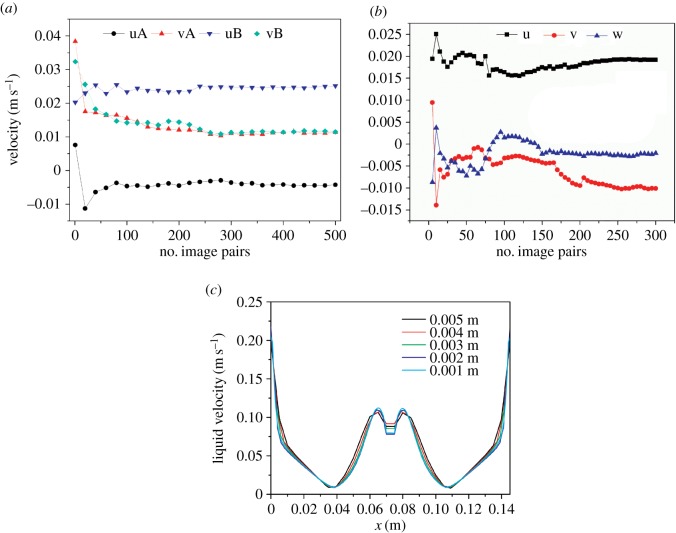
Study of the image pairs independence and grid independence: (*a*) PIV, (*b*) V3V and (*c*) mathematical model.

The statistical reliability of the number of PIV image pairs in the experimental flow field analysis was obtained by examining its effects on the *x-* and *y*-components of the mean liquid velocity. Such effects are demonstrated in figure 3*a* at two sample points A (*x* = 0.115, *y* = 0.105) and B (*x* = 0.115, *y* = 0.105), where the velocities and their fluctuation components appear to sufficiently converge after 300 image pairs. Although larger ensembles would be ideal, averaging a large number of image pairs to ensure statistical convergence was not feasible [27].

The statistical reliability analysis of the number of V3V image pairs was carried out using an identical approach. Three directions of the mean liquid velocity at a sample point are shown in figure 3*b*. The velocities and their fluctuation components appear to stabilize when the number of images is 300 pairs. In this work, 300 image pairs were acquired because the analysis of V3V data is a time-consuming process.

To ensure the quality of the simulation results, mesh independence tests were performed along different directions to determine whether a decrease in mesh size had a significant effect [55]. Five different quadrilateral element numbers from 1.0 × 10^3^ to 2.3 × 10^4^, which correspond to a mesh size of 0.005–0.001 m, were used in the mathematical model. The effects of the mesh size on the velocity profiles across line *y* = 0.105 m and *x* = 0.035 m are shown in figure 3*c*. The simulated velocity profiles were independent of the mesh size from 0.003 to 0.001 m. However, the velocity profiles showed an obvious deviation when the mesh size was larger than 0.003 m, whereas little improvement was achieved when the size was smaller than 0.003 m. As a result, a mesh size of 0.003 m was selected for all subsequent simulations.

### 3.2. Flow patterns of the three-dimensional three-component velocity field

**Figure 4.**
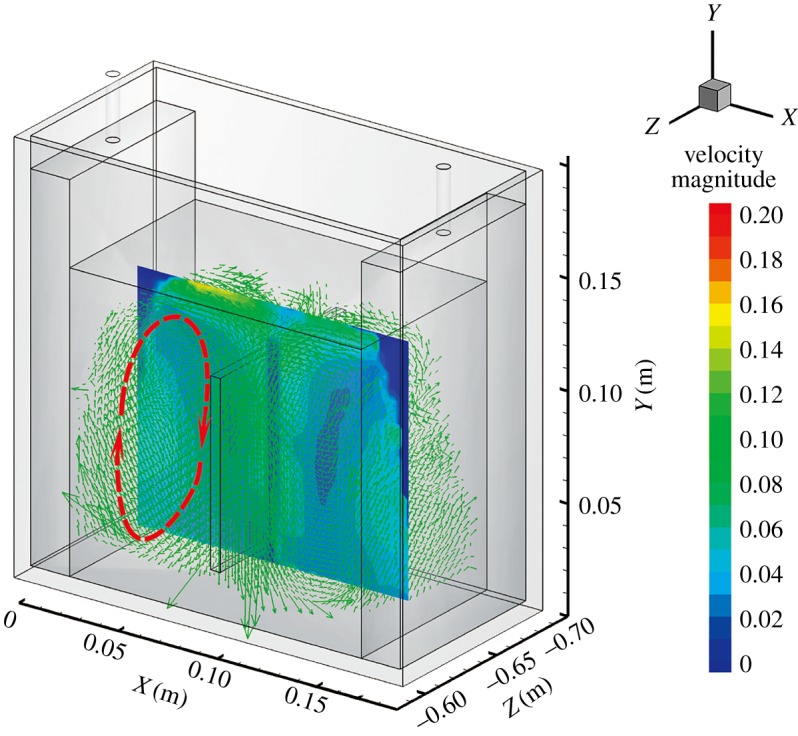
Three-dimensional vectors and a velocity contour slide of the mean liquid velocity field with a gas flow rate of 1.5 l min^−1^; these results were obtained using the V3V technique.

The instantaneous and time-averaged whole-field liquid velocities were simultaneously measured in the rectangular cell using the PIV and V3V techniques. The gas at the anode in the experiment was released at a constant flow rate of 1.5 l min^−1^. Figure 4 shows the details of the V3V-determined liquid velocity field with a 1.5 l min^−1^ gas flow rate, a velocity contour slide (*z* = −0.65 m) and vectors. This graph contains almost 11 000 three-dimensional spaced velocity vectors. They clearly show that the liquid flows were predominantly in the upward direction along the side of the anodes, because the liquid was dragged up by the rising bubbles released at the anode. The velocity vectors then turned 90° and moved towards the central cathode at a height from 0.10 to 0.14 m. Away from the anode and towards the cathode, the velocity vectors changed direction. The disadvantage of the PIV method is its ability to record only the projection of the velocity vector on the light sheet. The unknown third velocity component cannot be measured, which can significantly affect the measurement of the in-plane components.

**Figure 5.**
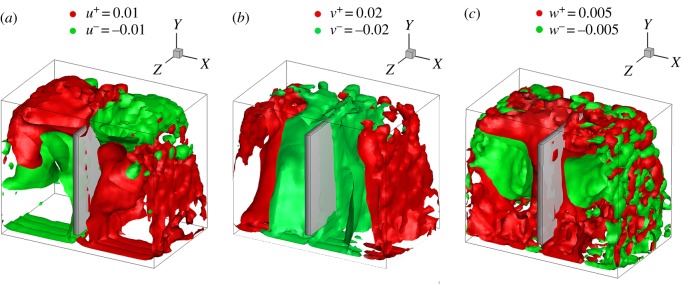
Isosurfaces of the mean liquid velocity components: (*a*) *u* = ±0.01 m s^−1^, (*b*) *v* = ±0.02 m s^−1^ and (*c*) *w* = ±0.005 m s^−1^.

Three-dimensional velocity data from the V3V experiments, including the *x*-, *y*- and *z*-components of the velocity (*u*, *v* and *w*), are shown in figure 5*a–c*, respectively, with different colours. In the isosurface plots, the positive and negative velocity components are shown in red and green, respectively. The magnitude of the velocity *v* in the direction of bubble motion was greater than the magnitudes of the other two velocity flow components. Velocity *w* in the *z*-direction was difficult to observe with the PIV data.

### 3.3. Effect of the flow rate on the gas release

**Figure 6.**
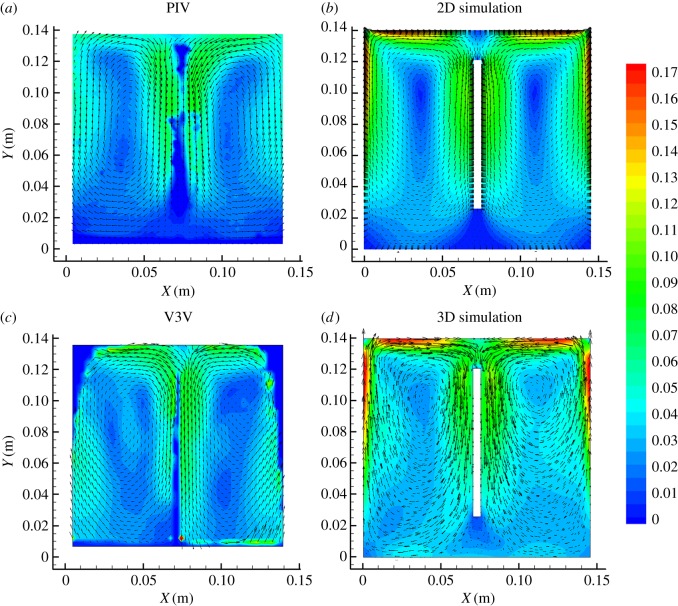
Comparison of the time-averaged flow field using the PIV and V3V techniques with 2D and 3D approaches for a gas flow rate of 1.5 l min^−1^: (*a*) PIV experiment, (*b*) 2D simulation, (*c*) V3V experiment and (*d*) 3D simulation.

Figure 6 compares the typical time-averaged velocity flow visualizations and numerical predictions across the centre plane (*z* = −0.65 m) for the gas inlet flow rate of 1.5 l min^−1^. As expected, the highest velocities occurred near the anodes. A pronounced double-vortex structure, which was centred in the upper part of the cell between the electrodes, was formed in the 2D experiment and computed instantaneous velocity fields. A similar flow structure evolved for the V3V measurements and 3D predictions. The velocity vectors were in the form of closed curves in the clockwise direction in the left channel (left of the cathode) and counter-clockwise direction in the right channel (right of the cathode). The computational results are consistent with the measurement results.

**Figure 7.**
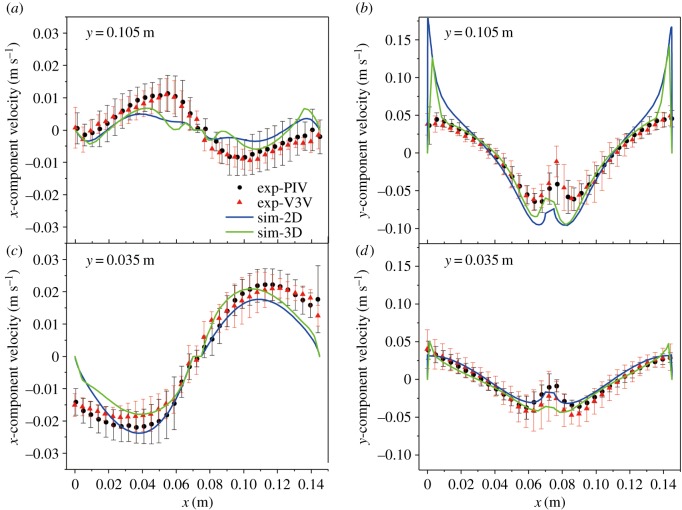
Comparison of the time-averaged velocity along different lines using the PIV/V3V techniques and 2D/3D approaches for a gas flow rate of 1.5 l min^−1^: (*a*) *u* along *y* = 0.105 m, (*b*) *v* along *y* = 0.105 m, (*c*) *u* along *y* = 0.035 m and (*d*) *v* along *y* = 0.035 m.

To quantitatively compare the experimentally determined velocity field with the model predictions, the velocity profiles were extracted from the time-average velocity fields. The velocity components along the horizontal lines *y* = 0.105 and 0.035 m in the centre plane were more desirable in this study. The PIV/V3V experiments were compared with the 2D/3D model predictions for a gas rate of 1.5 l min^−1^, as shown in figure 7. The dotted lines represent the experimental data, whereas the solid lines represent the corresponding simulation data. The error bars along the experimental curve were calculated from 10 sets of experimental results.

Figure 7*a* shows the variation of the time-averaged *x*-component of velocity *u* along the horizontal line *y* = 0.105 m. The profile of *u* was antisymmetrical: the left half was a positive hump, and the right side was a negative hump. Figure 7*b* shows that the profiles of the *y*-component *v* of the velocity along the two lines were symmetrical with respect to the central geometrical plane of the cell. Near the left and right walls, because of the drag effects, the velocity reached its maximum value. Near the cathode, the flow was primarily in the downward direction and the velocity was negative. The trend of *u* along *y* = 0.035 m was reversed and formed an s-shape, as shown in figure 7*c*. Moreover, the maximum velocity occurred in the middle of the channel, and the velocity magnitude decreased to zero near the left and right anodes, where the liquid was primarily vertical. As shown in figure 7*d*, the magnitude of the *y*-component *v* of the velocity substantially changed near the anode. The liquid velocity *v* along *y* = 0.105 m in figure 7*b* was larger than that along *y* = 0.035 m in figure 7*d*. This velocity component in the two channels was in the counter-clockwise and clockwise directions. The PIV and V3V measurements of the velocity near the anode were inaccurate because of the disturbance of the bubbles.

**Figure 8.**
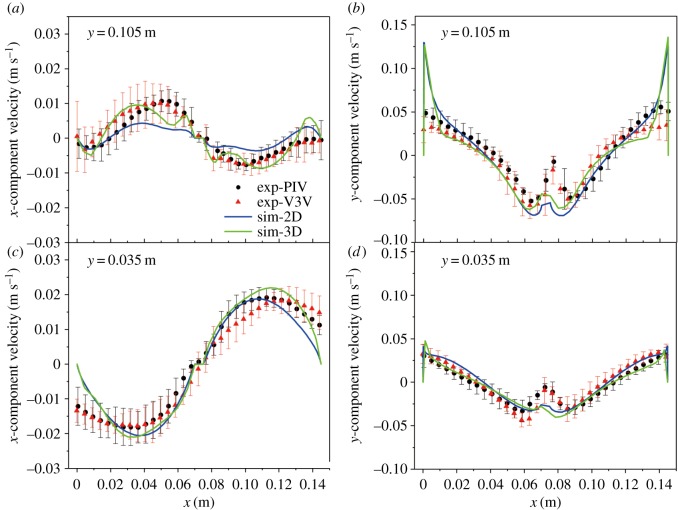
Comparison of the time-averaged velocity along different lines using the PIV/V3V techniques and 2D/3D approaches for a gas rate of 1.0 l min^−1^: (*a*) *u* along *y* = 0.105 m, (*b*) *v* along *y* = 0.105 m, (*c*) *u* along *y* = 0.035 m and (*d*) *v* along *y* = 0.035 m.

The same analysis was conducted for a gas flow rate of 1.0 l min^−1^ in the same experimental apparatus. Figure 8 shows the velocity components of the experimental measurements and model predictions. Again, the spatial variation of the velocity was obtained along *y* = 0.105 and 0.035 m; the dotted lines represent the experimental data, and the solid lines represent the corresponding simulation data. The consistency between experimental measurements and simulation predictions was also reasonable for the gas flow rate of 1.0 l min^−1^. No substantial change was observed in the trend of these velocity component profiles. However, the velocity magnitude was slightly smaller than that in figure 8 because of the smaller gas flow rate.

Figures 7 and 8 show that the velocity profiles obtained using the PIV and V3V techniques exhibited identical trends. The velocity magnitudes obtained from the PIV measurement were slightly different from those obtained using the V3V method under identical experimental conditions. The liquid motion predictions were consistent with the experimental data. However, some precision errors remained, particularly with the 2D model, and the velocities profiles appeared more smoothed. The motion in the region with high velocity was not accurately captured in the 2D simulation. The 3D model could capture more details of the flow behaviour, and both velocity components were accurately predicted by the simulations along the two horizontal lines.

**Figure 9.**
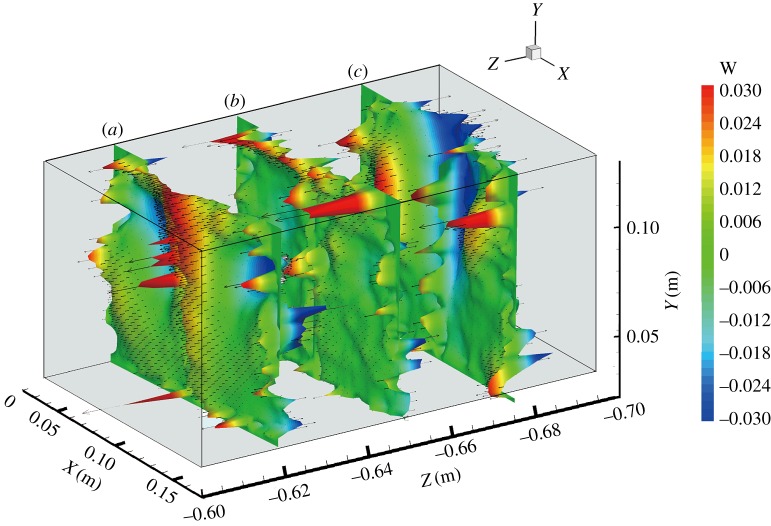
The *z*-component of the velocity vectors and contour of the liquid extracted from the V3V data: (*a*) *z* = −0.62 m, (*b*) *z* = −0.65 m and (*c*) *z* = −0.68 m.

To clearly show the key characteristics of the velocity distributions, the *z*-component of the velocity in three planes of *z* = −0.62, −0.65 m and −0.68 m (extracted positions from the V3V data) is shown in figure 9, and its *z*-axis was extended for better observation. The *z*-component of the velocity appeared higher near the anode and cathode (the blue and red regions). This result confirms that the flow in this system was 3D in nature.

**Figure 10.**
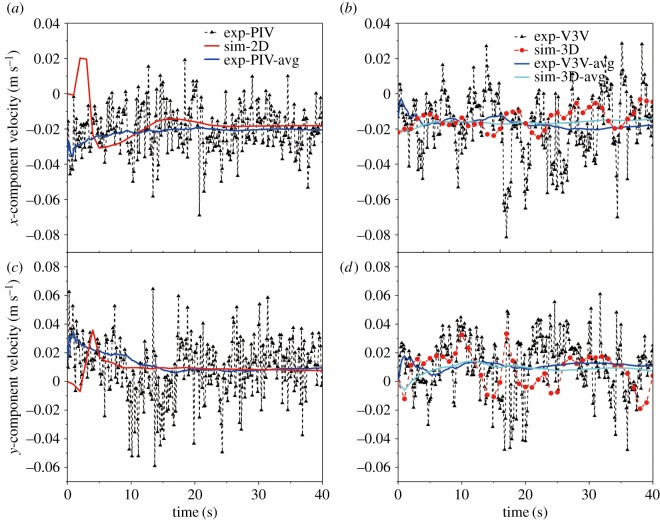
Comparison of the instantaneous velocity at point (0.11 m, 0.105 m, −0.65 m) using PIV/V3V techniques with 2D/3D approaches for a gas flow rate of 1.5 l min^−1^: (*a*) *u* using the PIV and 2D model, (*b*) *u* using the V3V and 3D model, (*c*) *v* using the PIV and 2D model and (*d*) *v* using the V3V and 3D model.

Three-component velocities at point (0.11 m, 0.105 m and −0.65 m) were extracted from the instantaneous velocity fields to analyse the flow field. Figure 10 compares the experimentally measured instantaneously fluctuating velocity in the time series with the 2D and 3D simulation predictions for a gas flow rate of 1.5 l min^−1^. The duration was 40 s, and its interval was 1/7 s. The velocities violently fluctuate with time because of turbulent fluctuations around a mean value. Because the gas flow rate in the experiment was constant, the temporal variation of the liquid velocity showed a steady state in the time average sense. Although the time-averaged velocity of the 2D model results was consistent with the experimental data, the predictions cannot reflect the fluctuation of the point velocity. The 3D model could capture the point velocity fluctuation, although its fluctuation magnitude was smaller than the experimental measurements. Hence, 3D model predictions are more suitable for the investigation in this system.

To further analyse the turbulence level produced by the bubble evolution, the turbulent intensity profiles were determined along the line *y* = 0.105 m for a gas flow rate of 1.5 l min^−1^. The turbulent intensity (*E*) was calculated from the root mean square of the turbulent fluctuation of the instantaneous liquid velocities as

3.1 $$E_{i}=\sqrt{\frac{1}{N}\sum_{n=1}^{N}{\left({U^{\prime }}_{i}\right)}^{2}}=\sqrt{\frac{1}{N}\sum_{n=1}^{N}{\left(U_{i}^{t}-{\bar{U}}_{i}\right)}^{2}},$$

where *U′* is obtained by subtracting the mean velocity components $\bar{\text{U}}$ from the instantaneous velocity *U*^*t*^; *i* = *x*, *y* and *z* represent the three directions.

The total turbulence intensity is

3.2 $$E_{\mathrm{total}}=\sqrt{\frac{1}{3}(E_{x}^{2}+E_{y}^{2}+E_{z}^{2})}.$$

**Figure 11.**
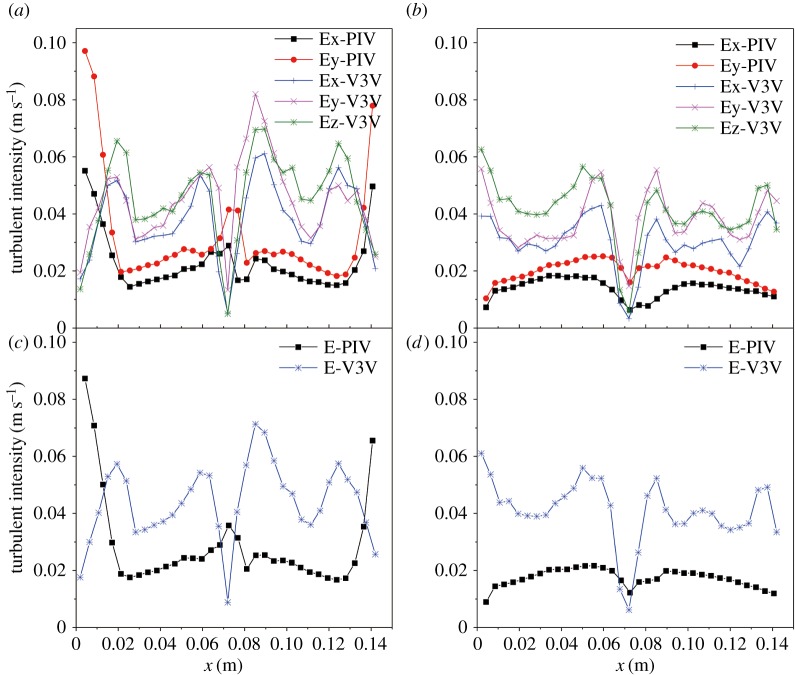
Comparison of the turbulent intensity using the PIV/V3V techniques with 2D/3D approaches for a gas flow rate of 1.5 l min^−1^: (*a*) *y* = 0.105 m, (*b*) *y* = 0.035 m, (*c*) total turbulent intensity along *y* = 0.105 m and (*d*) total turbulent intensity along *y* = 0.035 m.

Typical profiles of turbulent intensity along *y* = 0.105 and 0.035 m for the gas flow rate of 1.5 l min^−1^ are shown in figure 11. The magnitude obtained using the PIV technique was consistent with that obtained using the V3V technique. The turbulent intensity profiles were almost symmetrical along the horizontal lines. For the total turbulence intensity, the maximum value occurred near the anode and cathode, where velocity was higher. As expected, the total turbulent intensity and three-direction turbulent intensity along *y* = 0.105 m were higher than those along *y* = 0.035 m. The turbulent intensities in the *y*-direction were also slightly larger than those in the *x-* and *z*-directions, and the differences increased near the anode.

**Figure 12.**
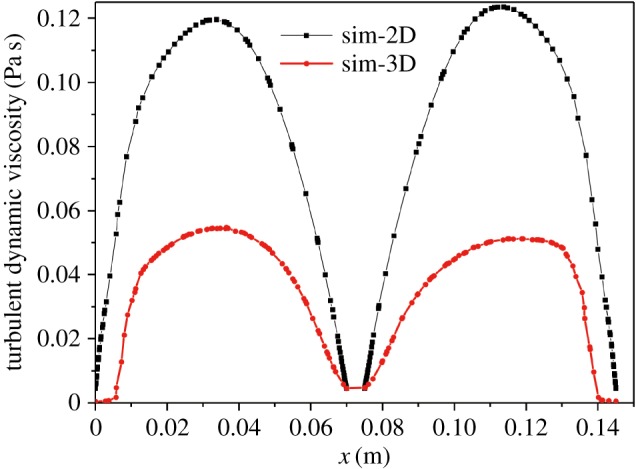
Comparison of the time-averaged velocity obtained using the 2D model and 3D approach for a gas flow rate of 1.5 l min^−1^ at *y* = 0.07 m.

The turbulent dynamic viscosity, which is an important parameter, was estimated to further investigate the mathematical modelling approach. The turbulent dynamic viscosity profiles in the 2D and 3D models along *y* = 0.07 m are shown in figure 12. The turbulent dynamic viscosity prediction obtained using the 3D approach was much lower than that obtained using the 2D model. This result is consistent with those reported by Ali & Pushpavanam [29]. The 2D model using the standard *k-ε* turbulence overestimates the effective viscosity value [49]. Only the predictions using the standard 3D turbulence model were consistent with the experiments.

## 4. Conclusion

A gas-evolving vertical electrode system was used to investigate the hydrodynamic characteristics of PIV and V3V techniques. Moreover, corresponding 2D and 3D computational fluid dynamics simulations were carried out using a two-fluid Euler–Euler model. Comparative analysis of the time-averaged flow field, three velocity components in the simulation data and experimental results showed that the mathematical model is reliable. The liquid velocity vectors were in the form of closed curves. The results of the V3V and 3D model also confirmed that the fluid flow in this channel of the gas-evolving electrode system is 3D in nature. V3V can be considered as a set of velocity vector multi-planes obtained using PIV and can accurately acquire the *z*-component of the velocity data in a 3D space. The *z*-component velocity in the V3V results was consistent with that in the 3D model prediction.

The instantaneous velocity in a time series at a point was considered and used to calculate the turbulent intensity, and the magnitude obtained by the PIV technique was consistent with that obtained by the V3V technique. However, the 2D predictions could not capture the fluctuation in the instantaneous velocity at the point and had a much lower turbulent dynamic viscosity than the 3D model. Hence, 3D model predictions are more suitable for investigating the gas-evolving vertical-electrode system.

## Data accessibility

This paper does not contain any additional data.
